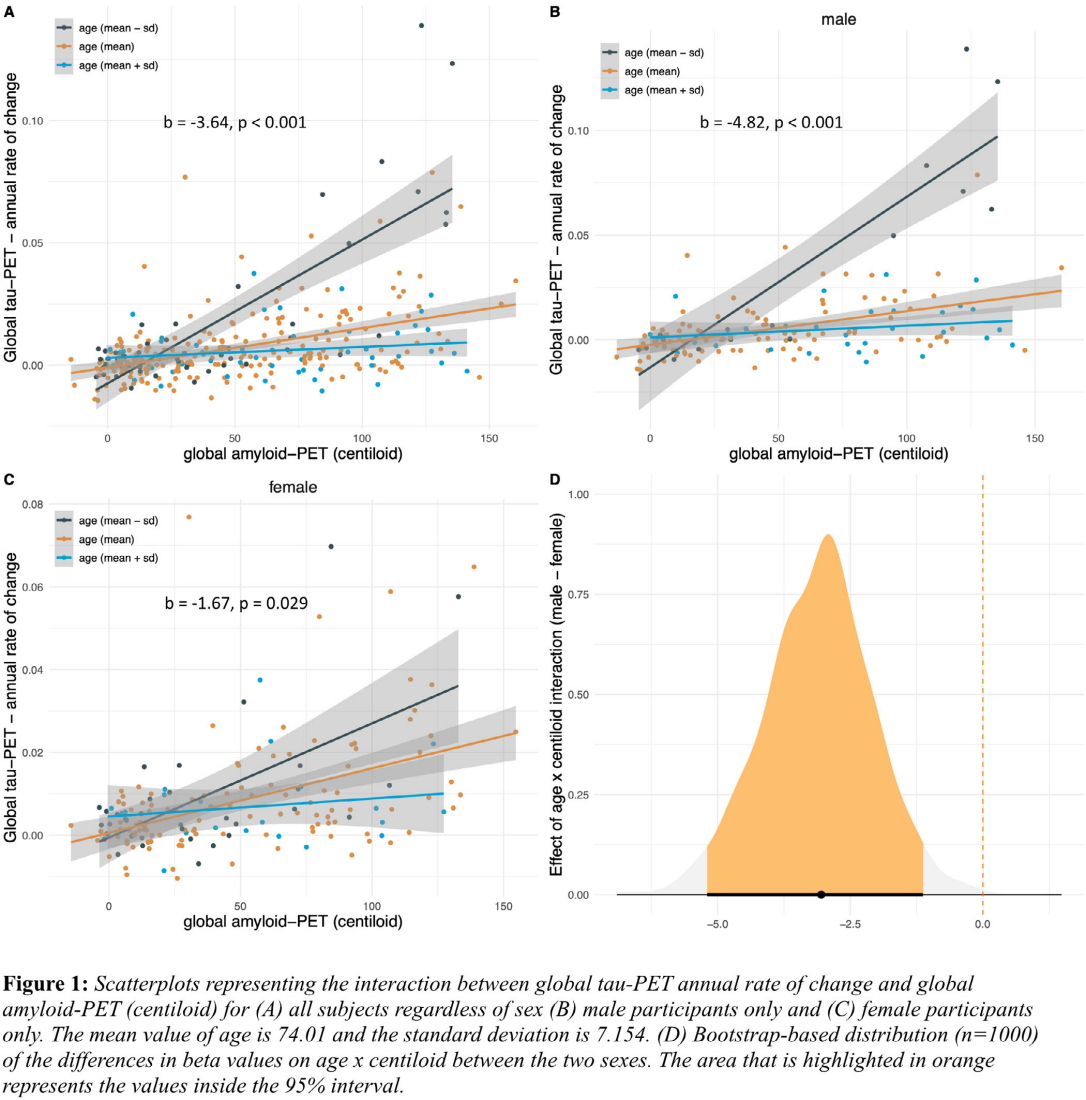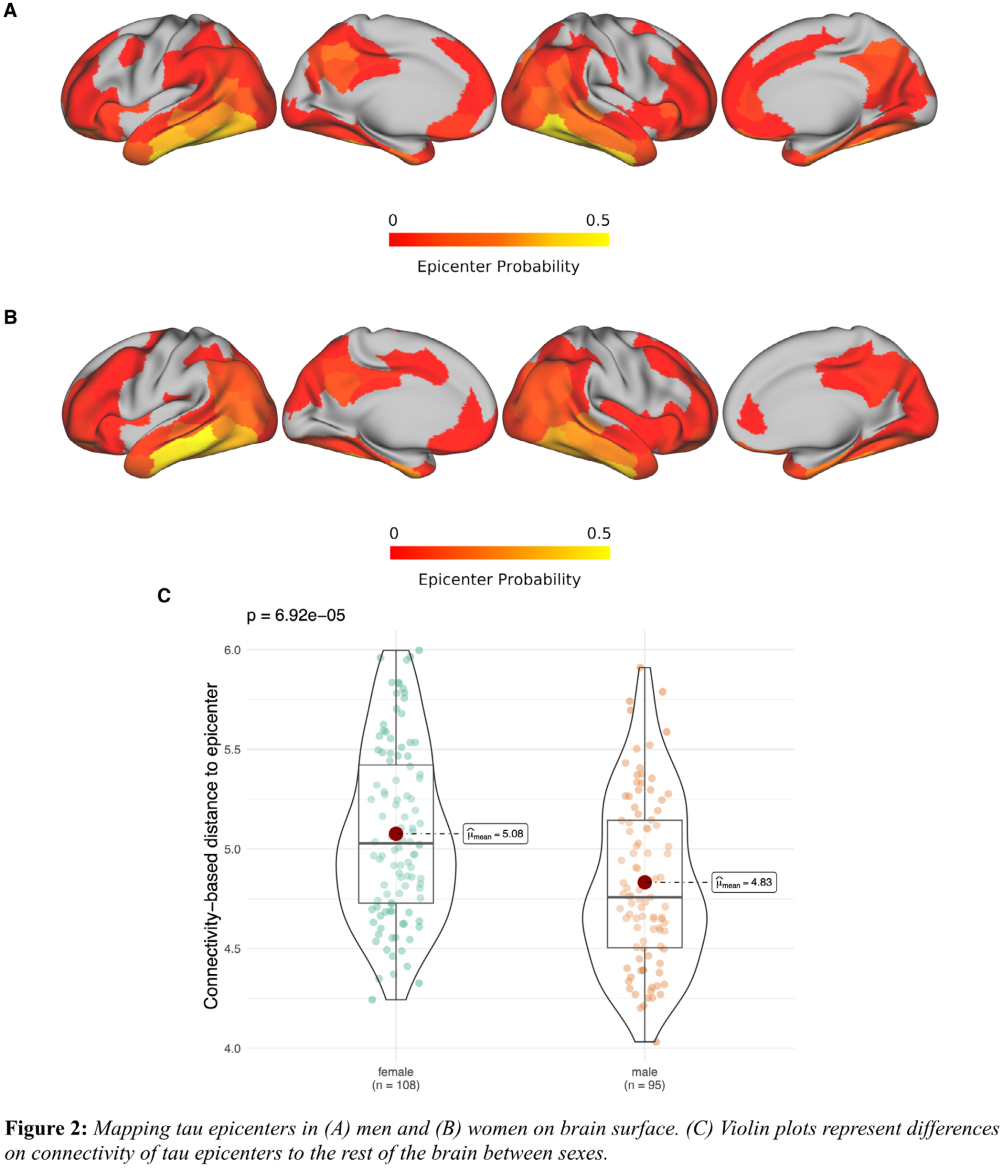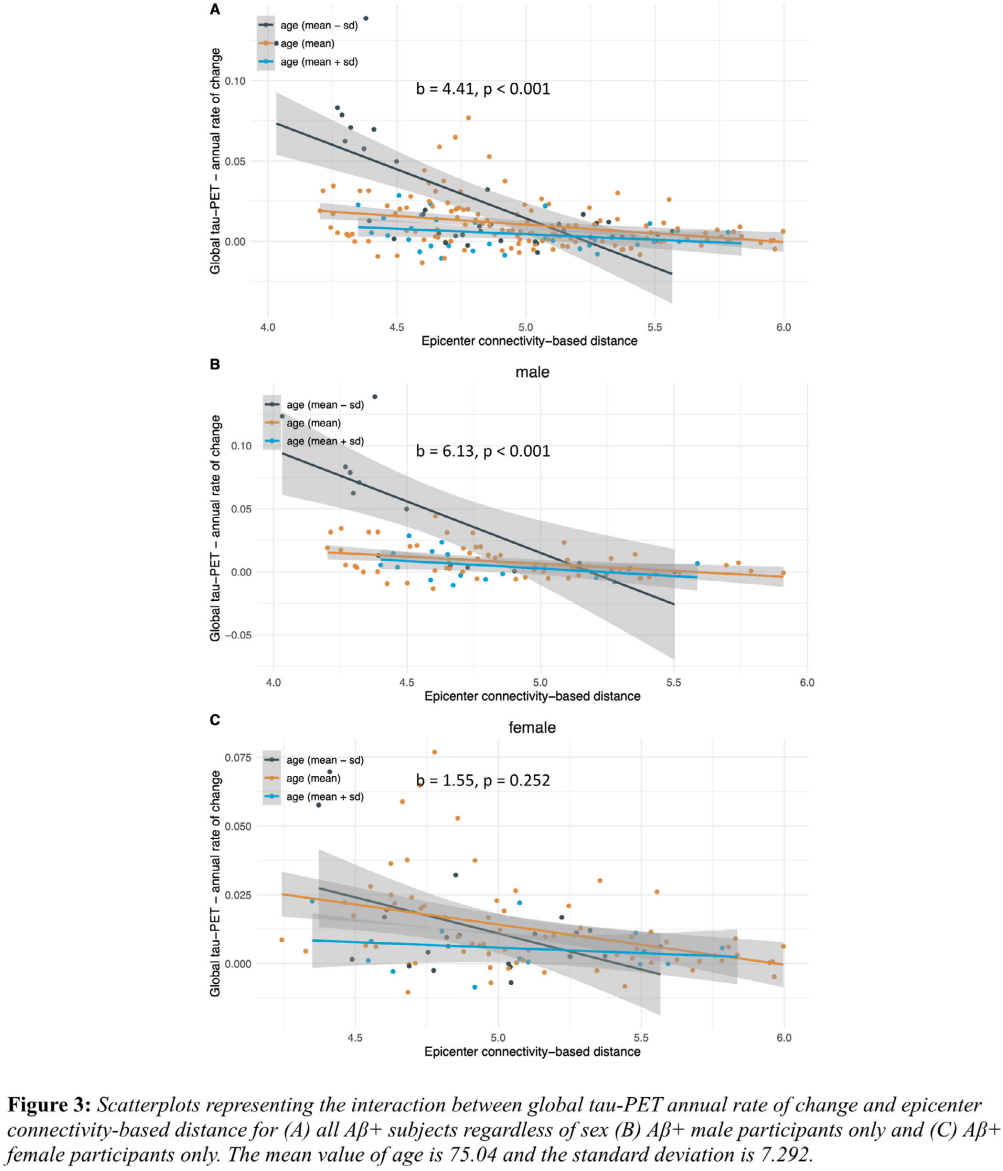# The association between the age and the rate of tau accumulation and spreading in different sexes

**DOI:** 10.1002/alz.090713

**Published:** 2025-01-03

**Authors:** Zeyu Zhu, Amir Dehsarvi, Sebastian Niclas Roemer, Anna Steward, Anna Dewenter, Mattes Gross, Fabian Wagner, Michael Ewers, Matthias Brendel, Nicolai Franzmeier, Alzheimer’s Disease Neuroimaging Initiative (ADNI)

**Affiliations:** ^1^ Institute for Stroke and Dementia Research (ISD), University Hospital, LMU, Munich, Bavaria Germany; ^2^ Department of Neurology, University Hospital, LMU, Munich, Bavaria Germany; ^3^ German Center for Neurodegenerative Diseases (DZNE), Munich, Bavaria Germany; ^4^ Institute for Stroke and Dementia Research (ISD), University Hospital, LMU, Munich Germany; ^5^ LMU University Hospital, Munich Germany; ^6^ Munich Cluster for Systems Neurology (SyNergy), Munich, Bavaria Germany; ^7^ University of Gothenburg, The Sahlgrenska Academy, Institute of Neuroscience and Physiology, Psychiatry and Neurochemistry, Gothenburg Sweden; ^8^ Institute for Stroke and Dementia Research (ISD), University Hospital, LMU, Munich, Bayern Germany

## Abstract

**Background:**

Neuroimaging studies have revealed age and sex‐specific differences in Alzheimer’s disease (AD) trajectories. However, how age and sex modulate tau spreading remains unclear. Thus, we investigated how age and sex modulate the amyloid‐beta (Aβ)‐induced accumulation and spreading of tau pathology from local epicenters across connected brain regions.

**Method:**

We included 313 ADNI participants (female/male, n = 167/146), i.e. 110 cognitively normal (CN) Aβ‐negative, and 203 Aβ‐positive subjects across the AD spectrum (i.e. CN/MCI/Dementia, n = 98/70/35) with baseline amyloid‐PET and longitudinal Flortaucipir tau‐PET. Annual tau‐PET change rates for 200 cortical regions of the Schaefer atlas were calculated. Sex‐specific resting‐state fMRI‐connectivity templates across the 200 Schaefer regions were determined in independent Aβ‐negative controls (female/male, n = 118/82) to determine the connectivity of tau epicenters to the rest of the brain. Using linear regression, we investigated interactions between age, sex and Aβ on tau accumulation and spread, controlling for APOE4‐status and diagnosis.

**Result:**

Higher Aβ (i.e. centiloid) predicted faster tau accumulation, where this association was pronounced in younger individuals (i.e. age x centiloid interaction, b = ‐3.64, p<0.001, Fig. 1A). This age x centiloid interaction was stronger in men (b = ‐4.82, p<0.001, Fig. 1B) vs. women (b = ‐1.67, p = 0.029, Fig. 1C), suggesting that younger age promotes Aβ‐related tau accumulation predominantly in men. Bootstrapping analysis further confirmed this effect (Fig. 1D). In Aβ+, epicenters with highest baseline tau‐PET showed a similar temporal‐lobe distribution in men and women (Fig. 2A&B), yet epicenter connectivity to the rest of the brain was stronger in men vs. women (Fig. 2C). Stronger connectivity of tau epicenters to the rest of the brain was linked to faster tau accumulation especially in younger Aβ+ subjects (i.e. interaction age x epicenter connectivity, b = 4.41, p<0.001, Fig. 3A). However, this effect was clearly driven by men (b = 6.13, p<0.001, Fig. 3B) and not observed when tested in women only (b = 1.55, p = 0.252, Fig. 3C).

**Conclusion:**

Aβ drives faster tau accumulation and this effect is particularly strong at younger age and even further pronounced in men, whose tau epicenters are more densely interconnected with the rest of the brain. Together, age and sex have clear modulating effects on tau spreading, and heterogeneous AD trajectories may be partly arisen due to sex‐specific differences in brain network architecture.